# Vancomycin-induced Henoch-Schönlein purpura: a case report

**DOI:** 10.1186/1752-1947-6-106

**Published:** 2012-04-10

**Authors:** Stanislas Bataille, Aurélie Daumas, Anne-Marie Tasei, Noémie Jourde-Chiche, Bertrand Dussol, Stéphane Burtey, Solène Taugourdeau, Yvon Berland, Laurent Chiche

**Affiliations:** 1 Centre de Néphrologie et Transplantation rénale, Hôpital de la Conception, Assistance Publique des Hôpitaux de Marseille, 147 Boulevard Baille, 13005 Marseille, Université Aix-Marseille II, France; 2 Laboratoire d'Anatomie Pathologique et Neuropathologie, Hôpital de la Timone, Assistance Publique des Hôpitaux de Marseille, 264 Rue Saint Pierre, 13385 Marseille, Université Aix-Marseille II, France; 3 Centre Régional de Pharmacovigilance, Hôpital Salvator, Assistance Publique des Hôpitaux de Marseille, 249 Boulevard Sainte-Marguerite, 13274 Marseille, Université Aix-Marseille II, France; 4 Service de Médecine Interne, Hôpital de la Conception, Assistance Publique des Hôpitaux de Marseille, 147 Boulevard Baille, 13005 Marseille, Université Aix-Marseille II, France

## Abstract

**Introduction:**

Henoch-Schönlein purpura is a small-vessel systemic vasculitis. Although its exact pathophysiology remains unknown, Henoch-Schönlein purpura has been reported in association with various medical conditions including hypersensitivity. We report the case of a patient with vancomycin-induced Henoch-Schönlein purpura.

**Case presentation:**

A 42-year-old Caucasian man who had previously undergone a heart transplant was diagnosed as having an intra-abdominal abscess after he underwent a Hartmann procedure. At 15 days after initiation of antibiotic therapy including vancomycin, he developed a purpuric rash of the lower limbs, arthralgia, and macroscopic hematuria. At that time, our patient was already on hemodialysis for end-stage renal disease. Henoch-Schönlein purpura was diagnosed. After a second 15-day course of vancomycin, a second flare of Henoch-Schönlein purpura occurred. Skin biopsies showed leucocytoclastic vasculitis with IgA deposits and eosinophils in the peri-capillary inflammatory infiltrate, suggesting an allergic mechanism. After vancomycin was stopped, we did not observe any further flares. Only five cases of isolated cutaneous vasculitis, one case of lupus-like syndrome and one case of Henoch-Schönlein purpura after vancomycin treatment have been described to date in the literature.

**Conclusions:**

Clinicians should be aware that systemic vasculitis can be induced by some treatments. Vancomycin is a widely prescribed antibiotic. Occurrence of rare but serious Henoch-Schönlein purpura associated with vancomycin requires its prompt discontinuation.

## Introduction

Henoch-Schönlein purpura (HSP) is a small-vessel systemic vasculitis. Its usual clinical presentation includes vascular purpura, abdominal pain, arthralgia, and glomerulonephritis. Although its exact pathophysiology remains unknown, HSP has been reported in association with various medical conditions such as cancer, blunt trauma, monoclonal IgA gammopathy, as well as in patients with Wiskott-Aldrich syndrome, chronic alcoholic liver disease, or α1-anti-trypsin deficiency [1]. HSP has also been described in association with hypersensitivity. Several drugs, such as ciprofloxacin, acetylsalicylic acid, carbidopa/levodopa, cocaine, acetyl cholinesterase inhibitors, carbamazepine and streptokinase have been involved in the induction of HSP [1]. Here, we report what is to the best of our knowledge only the second case of vancomycin-induced HSP.

## Case presentation

A 42-year-old Caucasian man was referred to our Nephrology Department. He presented with acute renal failure linked to severe dehydration secondary to diarrhea that had lasted a week. He was known to have end-stage renal disease secondary to nephrotoxicity from calcineurin inhibitors administered over 15 years as part of his heart transplant anti-graft rejection regimen, but had not started hemodialysis yet. His estimated glomerular filtration rate (GFR), using Modification of Diet in Renal Disease (MDRD) calculation, was 16.8 mL/minute/1.73 m^2 ^before admission, and he had only mild proteinuria (0.56 g/24 hours) and no hematuria.

On admission, his clinical and specifically his abdominal examinations were unremarkable except for the presence of non-hemorrhagic and non-mucoid diarrhea associated with fever. Community-acquired infectious gastroenteritis was suspected. Our patient's medical history revealed he had been diagnosed as having a transposition of the great arteries that led to an initially successful heart transplantation 15 years ago, before the occurrence of chronic cardiac transplant rejection. As a former smoker, he had also had an isolated ischemic stroke five years ago, but had no history of allergies or anaphylaxis. A recent echocardiograph showed congestive heart failure, with, a left ventricular ejection fraction of 30% and pulmonary hypertension. His immunosuppressive regimen included cyclosporin (45 mg twice a day) and prednisone (5 mg/day). He was given intravenous antibiotic therapy including ceftriaxone (1 g/day) and oral ciprofloxacin (500 mg/day).

**Figure 1:**
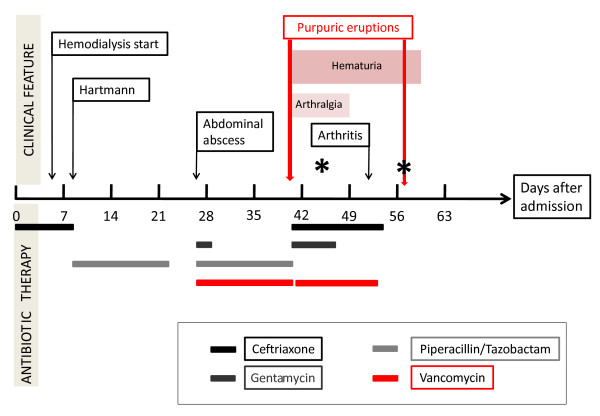
**Timing of administration of various antibiotics and occurrence of signs related to Henoch-Schönlein purpura**. *Skin biopsy.

Because he presented with symptoms of heart failure following rehydration, as well as no improvement of his renal function, intermittent hemodialysis was started on day five after admission after the placement of a temporary jugular catheter. By day nine, because of persistent sepsis and diarrhea despite the antibiotic therapy, an abdominal computed tomography (CT) scan was performed and our patient was diagnosed as having stercoral peritonitis secondary to a colonic diverticular perforation. *Bacteroides fragilis *was identified in blood cultures. A Hartmann surgical procedure was performed and the antibiotic therapy was switched to piperacillin/tazobactam (4 g twice a day) (Figure 1).

By day 24 after admission, our patient showed clinical improvement. He had no further fever or diarrhea, and his biological test results had normalized, so piperacillin/tazobactam was stopped. On day 27, his fever recurred. A second abdominal CT scan showed an abdominal peri-hepatic abscess, and antibiotic therapy was restarted (piperacillin/tazobactam (4 g twice a day), gentamycin (a single injection), and vancomycin (one injection at each hemodialysis session). By day 36, our patient became afebrile and a third CT scan showed regression of the peri-hepatic fluid collection. Antibiotic therapy was again stopped on day 41, after a second 15-day course.

**Figure 2:**
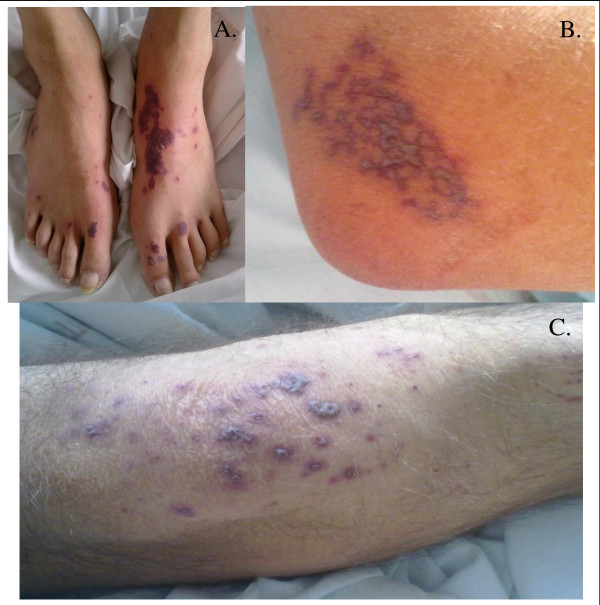
**Image of the first necrotic purpura eruption on the feet (A), elbows (B), and knees (C)**.

**Figure 3:**
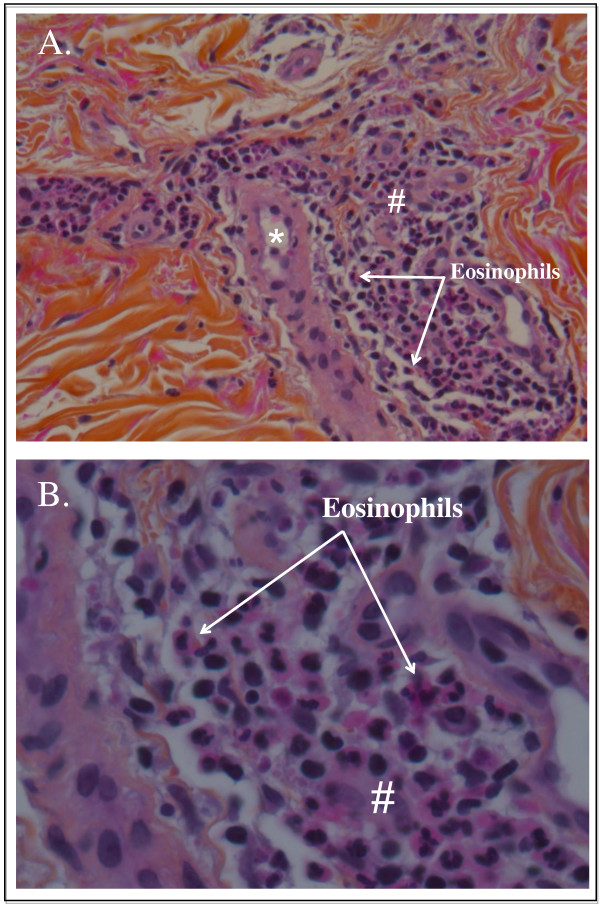
**(A, B) The second biopsy (performed a few hours after the second purpuric eruption), showing dermal leucocytoclastic vasculitis with eosinophils deposits suggestive of vancomycin-induced Henoch-Schönlein purpura**. #Peri-vascular inflammatory cells; *dermal capillaries.

On the day the antibiotic therapy should have been stopped, our patient had necrotic purpura eruptions on the external sides of his feet, knees, and elbows (Figure 2). Concomitant with the purpuric eruptions, he also presented with arthralgia and macroscopic hematuria, but remained afebrile. Arthralgia was bilateral, and located in the joints near the purpuric eruptions. Hematuria was asymptomatic, and was associated to either other urinary signs, or intra-vesical clotting. This new flare was accompanied by systemic inflammatory response syndrome and elevated C-reactive protein level (124 mg/L; normal values < 3 mg/L), and a new empirical antibiotic therapy was initiated, including intravenous gentamycin, ceftriaxone, and valaciclovir. Vancomycin was continued. An echocardiograph showed no signs of endocarditis. Blood cultures, urine analysis, as well as polymerase chain reaction testing for herpes simplex virus 1 (HSV1), HSV2, and varicella zoster virus (VZV), and serology for HSV1, HSV2, VZV, Epstein-Barr virus (EBV), human herpesvirus 6 (HHV6), human herpesvirus 8 (HHV8), cytomegalovirus (CMV), hepatitis B virus (HBV), hepatitis C virus (HCV), human immunodeficiency virus (HIV), parvovirus B19, rickettsia, and Lyme disease, did not show any evidence of recent viral, bacterial, or fungal infections. Differential diagnostic investigations included tests for anti-nuclear antibodies, cryoglobulin, cryofibrinogen, complement dosage, protein electrophoresis, immunoglobulin dosage; results were positive for isolated anti-nuclear antibody (1/200, with no specificity), and increased circulating polyclonal IgA level (4.71 g/L; normal values: 0.88 to 4.1 g/L). Skin biopsies, performed four days after the eruptions, showed leucocytoclastic necrotic vasculitis of the dermal capillaries (Figure 3), with neutrophils and lymphocytes in and around the blood vessels and intra-vascular fibrin thrombi, all of which are characteristic features of vasculitis. Immunofluorescence examination showed IgM and C3 deposits in the dermal capillary walls.

On day 53 after admission, that is, 12 days after the first eruption occurred and after antibiotic therapy was restarted, our patient presented with arthritis of the right knee and left elbow. Arthritis regressed spontaneously within 48 hours, and no articular puncture was performed.

On day 58 after admission, he then presented with a second purpuric eruption of both legs, associated with arthralgia and macroscopic hematuria. All bacteriological and viral test results were negative and he was still receiving antibiotics at that time, so that antibacterial therapy was interrupted. A full-body CT scan showed no more abscess and no adenopathy, but ascitis, which on puncture produced a sterile fluid. A second skin biopsy was performed on the day the purpura appeared. It showed the same leucocytoclastic dermal vasculitis with eosinophils, but vascular IgA and C3 deposits were found with immunofluorescence imaging (Figure 3).

After antibiotic therapy was stopped, our patient finally recovered entirely with disappearance of purpura, arthritis and hematuria, and has not relapsed after a follow-up period of eight months.

## Discussion

HSP is a small-vessel systemic vasculitis characterized by vascular wall deposits of immune complexes containing mainly IgA. It can affect the skin, gut, and glomeruli vessels, and is often associated with purpura, abdominal pain, hematuria, and arthralgia or arthritis [1]. HSP is the most common vasculitis in childhood, but can also occur in adults [1].

Diagnostic criteria for HSP in children have been recently validated as an association between the presence of purpura in a lower limb plus one of the following four criteria: (1) abdominal pain, (2) histopathology (that is, IgA), (3) arthritis or arthralgia, and (4) renal involvement [2].

In our patient, concomitant occurrence of purpuric lesions, arthritis, macroscopic hematuria, and an exhaustive immunological exploration with negative results confirmed the diagnosis of HSP. Consistent with this diagnosis, his plasmatic IgA dosage was elevated.

A very surprising finding was the involvement of the kidney in our patient with end-stage renal disease. The chronology was clearly suggestive of HSP, with, macroscopic hematuria occurring concomitantly to each new purpuric eruption, and disappearing within a few days. However, as he was already under hemodialysis, we were not able to fully evaluate the severity of the renal involvement of the vasculitis in our patient.

In our patient, the first skin biopsy, performed several days after the purpuric eruption, showed leucocytoclastic vasculitis and IgM deposits (by immunofluorescence) (Figure 3). Specific leucocytoclastic vasculitis of the skin is a main symptom of HSP [1]. It is classically associated with IgA deposits, but might also be associated, as in our patient, with IgM deposits, especially when a biopsy is performed later, and when the kidney is involved [3]. Indeed, the second biopsy, performed shortly after the eruption, showed IgA deposits (by immunofluorescence), which confirmed HSP. Consistent with an allergic mechanism, optical microscopy showed the presence of eosinophilic polynuclear cells in the inflammatory infiltrate.

The pathophysiology of HSP has not been totally elucidated, but may be related to the production of abnormally glycosylated IgA, which is not sufficiently cleared by the liver and leads to the formation of IgA macromolecules that accumulate in the circulation, and subsequently become deposited in the vessel walls causing vasculitis [1]. Most cases of HSP are primary, but some episodes can be related to an identified etiology, such as infection, medication [4], or neoplasia [1].

Our patient had no history of HSP. Thus, it was probably secondary to his recent medical condition. An infectious cause was unlikely as our patient had no fever at the time of the first cutaneous eruption; no bacterial, fungal or viral infection was found, although extensive explorations were made, and the purpuric eruption relapsed while our patient was receiving an antibiotic therapy and the intra-abdominal abscess was well controlled on the CT scans. Further, purpura appeared after antibiotic therapy initiation and disappeared after antibiotic therapy withdrawal. Finally, eosinophils were found in skin biopsies.

From the literature, drugs that have already been associated with HSP are clarithromycin, carbidopa, cytarabine, enalapril/lisinopril, ciprofloxacin, acetylsalicylic acid, cocaine, acetyl cholinesterase inhibitors, carbamazepine, and streptokinase [1].

In our patient's case, the evolution of HSP was strongly linked with vancomycin: both attacks occurred about 15 days after vancomycin treatment was started, and after vancomycin was stopped the evolution of HSP stopped and our patient was cleared of HSP. In the literature, vancomycin-induced vasculitis has been reported to occur from 1 to 27 days after the start of vancomycin therapy [4-9].

According to a French causality assessment method for unexpected or toxic effects of drugs [10], chronological evolution is very suggestive (first attack 16 days after treatment with vancomycin was started, aggravation after reintroduction, and then regression of symptoms with no more HSP attacks after vancomycin was stopped) when there is no evidence of other possible etiologies.

Several cases of vasculitis following vancomycin treatment have been reported in the literature: five cases of isolated leucocytoclastic cutaneous vasculitis [5-8] and one case of lupus-like syndrome [9]. To the best of our knowledge, only one case of HSP linked to vancomycin has been described previously [4]. In that case, a patient treated with vancomycin with a staphylococcal chest infection presented with HSP and histologically proven acute interstitial nephritis. As he had preserved renal function, the interstitial nephritis was associated with oliguric acute renal failure. The authors hypothesized that either an anaphylactic reaction or a staphylococcal glycoprotein release had initiated HSP.

No other cases were found in the French Pharmacovigilence database using the keywords HSP, vancomycin, or teicoplanin. Interestingly, Marshall *et al*. reported induced vasculitis cross-reactivity between teicoplanin and vancomycin [7].

## Conclusions

We report only the second case of vancomycin-induced Henoch-Schönlein purpura that involved the skin, joints, and kidneys. Diagnosis was made possible by the combination of specific clinical and histological findings, and in particular the timing of events with regard to the introduction and withdrawal of glycopeptides. This is a rare, maybe under-diagnosed, but serious adverse event that needs to be considered and if suspected, should lead to prompt discontinuation of glycopeptides.

This case has been registered as French Pharmacovigilence case no. MA20110866; Base Nationale de Pharmacovigilance.

## Consent

Written informed consent was obtained from the patient for publication of this manuscript and any accompanying images. A copy of the written consent is available for review by the Editor-in-Chief of this journal.

## Competing interests

The authors declare that they have no competing interests.

## Authors' contributions

SB, AD, NJC, BD and SB are the clinicians of our hemodialysis unit, intensive care unit and nephrology department who treated our patient during the whole hospitalization. Each played an important part in the treatment of this unusual case. AMT performed histological examination of the skin biopsies. ST is a pharmacologist and studied the different possibilities of therapeutic etiologies of Henoch-Schönlein purpura. She was also a major contributor in writing the manuscript. YB is the department chief. LC is an internist; he first brought up the possibility of vancomycin-induced Henoch-Schönlein purpura and was a major contributor in writing the manuscript. All authors have read and approved the final manuscript.
